# Effects of Na^+^ channel blockers on the restitution of refractory period, conduction time, and excitation wavelength in perfused guinea-pig heart

**DOI:** 10.1371/journal.pone.0172683

**Published:** 2017-02-23

**Authors:** Oleg E. Osadchii

**Affiliations:** 1 Department of Biomedical Sciences, University of Copenhagen, Copenhagen, Denmark; 2 Department of Health Science and Technology, University of Aalborg, Aalborg, Denmark; University of Minnesota, UNITED STATES

## Abstract

Na^+^ channel blockers flecainide and quinidine can increase propensity to ventricular tachyarrhythmia, whereas lidocaine and mexiletine are recognized as safe antiarrhythmics. Clinically, ventricular fibrillation is often precipitated by transient tachycardia that reduces action potential duration, suggesting that a critical shortening of the excitation wavelength (EW) may contribute to the arrhythmic substrate. This study examined whether different *I*_Na_ blockers can produce contrasting effects on the rate adaptation of the EW, which would explain the difference in their safety profile. In perfused guinea-pig hearts, effective refractory periods (ERP), conduction times, and EW values were determined over a wide range of cardiac pacing intervals. All *I*_Na_ blockers tested were found to flatten the slope of ERP restitution, indicating antiarrhythmic tendency. However, with flecainide and quinidine, the beneficial changes in ERP were reversed owing to the use-dependent conduction slowing, thereby leading to significantly steepened restitution of the EW. In contrast, lidocaine and mexiletine had no effect on ventricular conduction, and therefore reduced the slope of the EW restitution, as expected from their effect on ERP. These findings suggest that the slope of the EW restitution is an important electrophysiological determinant which can discriminate *I*_Na_ blockers with proarrhythmic and antiarrhythmic profile.

## Introduction

Na^+^ channel blockers are class I antiarrhythmic agents which may suppress tachyarrhythmia by prolonging the effective refractory period (ERP) and therefore eliminating the excitable gap in re-entrant circuit. Nevertheless, the clinical experience in using these agents to control cardiac arrhythmia has been challenged by observations on ventricular proarrhythmic responses that may occur in 1–8% of patients receiving Na^+^ channel blocker therapy [1–2]. In this regard, the Cardiac Arrhythmia Supression Trial (CAST) has demonstrated that administration of flecainide, class Ic agent, to patients with healed myocardial infarction was associated with a 2.5-fold greater risk of sudden arrhythmic death as compared to patients treated with placebo [3]. Flecainide is therefore currently contraindicated in patients with structural heart disease. Quinidine, class Ia agent previously used to restore sinus rhythm in atrial fibrillation, has been shown to facilitate the life-threatening ventricular tachyarrhythmia and increase cardiac mortality rates over long-term follow-up [4–5]. Accordingly, quinidine is not generally used now for antiarrhythmic therapies, excepting some special clinical conditions (e.g. Brugada syndrome). In contrast, class Ib Na^+^ channel blockers such as lidocaine and mexiletine are thought to be safe antiarrhythmics [6–7]. The mechanisms contributing to the striking difference in arrhythmic safety profile between different subgroups of class I agents remain incompletely understood. It is noteworthy, however, that in contrast to class Ia and Ic agents which slowly dissociate from the Na^+^ channel and hence significantly delay ventricular conduction, lidocaine and mexiletine exhibit fast kinetics of the *I*_Na_ blockade, and produce little or no change in conduction velocity in non-ischemic hearts [8–9].

Ambulatory ECG monitoring in victims of sudden death suggests that fatal cardiac arrest is typically preceded by abrupt heart rate acceleration leading to the reduction in ventricular action potential duration (APD) [10–11]. These observations imply that ventricular fibrillation (VF) may be precipitated by the critical shortening of the excitation wavelength, the distance traveled by the activation front during the duration of the refractory period. Upon an excessive shortening of the excitation wavelength, the area of depolarized cells could be reduced to such an extent that the available current strength can become insufficient in order to depolarize downstream myocytes. This would translate to the source-to-sink mismatch and cause conduction failure [12–13]. The localized conduction block then can provoke fragmentation of the propagating wavefront, thus leading to highly disorganized electrical activation, a hallmark of VF. In support of this mechanism, an increase in the slope of APD rate adaptation (the electrical restitution) has been demonstrated to increase the propensity to develop VF, whereas flattening the restitution slope was proved to be antiarrhythmic [14–15].

These considerations suggest that drug-induced changes in the rate adaptation of the cardiac excitation wavelength can be critical for understanding the difference in arrhythmic safety profile among different subgroups of the Na^+^ channel blockers. The present study, therefore, was designed to test the hypothesis that the excitation wavelength restitution can be steepened by flecainide and quinidine, whereas lidocaine and mexiletine can reduce the electrical restitution slope. The hypothesis was proposed by considering the following arguments. The ventricular excitation wavelength can be found as a product of effective refractory period (ERP) and conduction velocity. Because an increase in cardiac activation rate leaves less diastolic time for *I*_Na_ recovery from drug-induced block, class I agent effects on ERP and ventricular conduction are likely to be use-dependent [8–9], i.e. more prominent at the short as compared to the long diastolic intervals. The use-dependent prolongation of refractoriness by Na^+^ channel blocker would then contribute to flattening the ERP restitution, indicating the antiarrhythmic tendency. With class Ib agents (lidocaine and mexiletine), this change may prevent an excessive shortening of the excitation wavelength upon heart rate acceleration, and therefore eliminate the substrate for VF. In contrast, with class Ia (quinidine) and Ic (flecainide) agents, the beneficial changes in ERP are likely to be eliminated owing to the marked conduction slowing at short diastolic intervals, an effect which can facilitate proarrhythmia via the mechanisms related to increased steepness of the excitation wavelength restitution. In the present study, these possibilities were examined by assessing the rate adaptation of ERP, ventricular conduction times, and the excitation wavelength in perfused guinea-pig hearts upon infusion of flecainide, quinidine, lidocaine and mexiletine.

## Materials and methods

This study complies with the European Community Guidelines for the Care and Use of Experimental Animals, and was approved by the Animal Ethics Screening Committee of the Panum Institute (clearance number: 2010/561-1799).

### Isolated, langendorff-perfused heart preparations

The experiments on isolated, perfused hearts were performed as described previously [16–17]. Female Dunkin-Hartley guinea-pigs weighing 400–500 g were anesthetized with sodium pentobarbital (50 mg/kg i.p.) and anticoagulated with heparin (1000 IU/kg i.p.). The chest was opened, the hearts were immediately excised, mounted on a Langendorff perfusion set-up (Hugo Sachs Elektronik-Harvard Apparatus GmbH, March-Hugstetten, Germany) and perfused via the aorta at a constant flow (15 ml/min) with carefully filtered, warmed physiological saline solution saturated with 95%O_2_ and 5%CO_2_. The perfusion solution contained (in mM) 118.0 NaCl; 4.7 KCl; 2.5 CaCl_2_; 25 NaHCO_3_; 1.2 KH_2_PO_4_; 1.2 MgSO_4_; and 10.0 glucose, and had a pH of 7.4. The aortic perfusion pressure (65–70 mm Hg) was measured with a ISOTEC pressure transducer and the coronary flow rate was determined using an ultrasonic flowmeter probe (Transonic Systems Inc., USA) placed just above the aortic cannula. The electrical activity of the heart preparations was assessed from the volume-conducted ECG as well as monophasic action potential recordings. Throughout the experiments, the heart preparations were kept immersed in the temperature-controlled, perfusate-filled chamber to minimize thermal loss. Aortic pressure, coronary flow rate, ECG and ventricular monophasic action potentials were continuously monitored using the 16-channel PowerLab system (ADInstruments, Oxford, UK).

### Electrophysiological recordings

**Fig 1:**
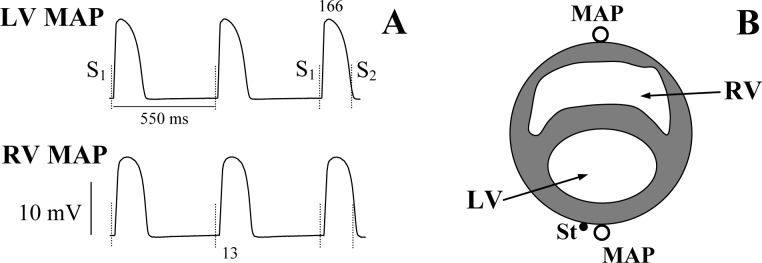
Representative monophasic action potential recordings obtained upon measuring the effective refractory period. Panel A shows monophasic action potentials (MAP) recorded from the left ventricular (LV) and the right ventricular (RV) epicardium upon extrasystolic stimulations applied at a basic drive cycle length of 550 ms. The moments of S_1_ and S_2_ application are shown by vertical dotted lines. The numbers (ms) shown indicate the effective refractory period (last beat in LV MAP) and the LV-to-RV conduction time (second beat in RV MAP). Panel B illustrates location of the LV and RV MAP recording electrodes (open circles), and the LV stimulating (St) electrode (filled circle).

In order to slow the intrinsic beating rate and enable ventricular pacing over a wide range of diastolic intervals, both atria were removed and the atrioventricular (AV) node was crushed mechanically with forceps prior to taking electrophysiological recordings. Monophasic action potentials (MAP) [18] were recorded at the left ventricular (LV) and right ventricular (RV) epicardial base (Fig 1) using J-shaped, spring-loaded pressure contact electrodes (Hugo Sachs Electronik-Harvard Apparatus GmbH, March-Hugstetten, Germany). The MAP duration was measured at 90% repolarization (APD_90_). Electrical stimulations were applied at LV epicardium close to the MAP recording site using 2 ms rectangular pulses of twice diastolic threshold current generated by a programmable stimulator (Hugo Sachs Electronik-Harvard Apparatus GmbH, March-Hugstetten, Germany). Epicardial pacing thresholds were measured both at baseline and upon drug infusion, and the stimulating current strength was adjusted appropriately whenever necessary.

The LV-to-RV conduction time during LV pacing was determined as the delay between the fastest upstroke of the LV action potential and the RV MAP recording. The effective refractory period was measured by application of the premature extrastimulus (S_2_) after a train of 20 regular (S_1_) pulses. The S_1_-S_2_ coupling stimulation interval was reduced from 210 ms in 5–10 ms steps until getting no capture. The ERP was defined as the longest S_1_-S_2_ interval producing no extrasystolic response (Fig 1). The relative excitation wavelength (EW) was calculated as a ratio of ERP and the LV-to-RV conduction time, as described previously [19–22], and presented throughout the paper as a dimensionless numerical variable.

### Electrical restitution

During extrasystolic stimulations, ventricular conduction times, ERPs, and the corresponding relative exctitation wavelength values were determined over a wide range of S_1_-S_1_ intervals in a basic drive train. The measurements were started with a S_1_-S_1_ cycle length of 550 ms (the longest pacing interval producing no ventricular escape beats), which was then reduced to 500 ms, followed by further reductions in steps of 20 ms over an S_1_-S_1_ range from 500 to 200 ms, and by 5–10 ms reductions from 200 ms down to pacing intervals (about 170 ms) producing 2:1 conduction block. In AV-blocked preparations, the minimum pacing intervals achieved with this protocol remain significantly greater than the threshold value of S_1_-S_1_ interval for inducing repolarization alternans (90–100 ms in intact guinea-pig heart upon normokalemic perfusion) [23], meaning that the whole dynamic pacing protocol could have been completed without inducing electrical instability.

**Fig 2:**
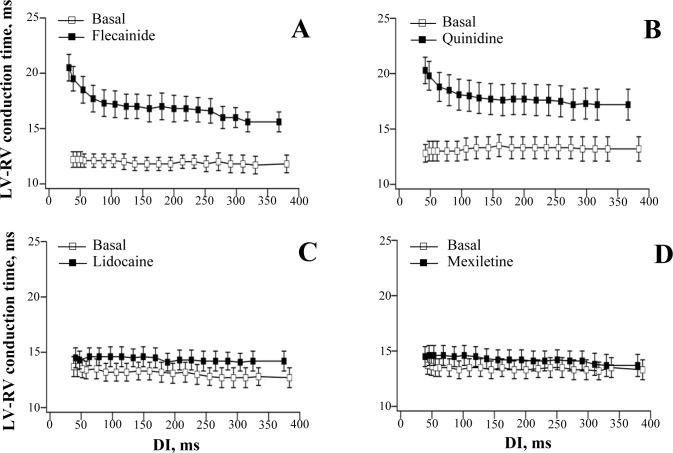
**Effects of flecainide (panel A), quinidine (panel B), lidocaine /panel C), and mexiletine (panel D) on LV-to-RV conduction time.** Conduction times were determined in the last beat of a train of 20 regular (S_1_) pulses during ERP measurements at baseline and following drug infusion. The S_1_-S_1_ interval in a train was progressively reduced from 550 ms to 170 ms, as described in Methods, to generate a range of diastolic intervals (DI) from about 380 ms to 40 ms. The heart was allowed to beat spontaneously for 5–10 s in between successive stimulations.

Once the electrical stimulations were performed, appropriate diastolic intervals (DI) were calculated as a difference between the S_1_-S_1_ cycle length and the ERP measured. The electrical restitution was assessed by plotting ERPs, conduction times, and the relative excitation wavelength as a function of the preceding DI [24]. The ERP and EW restitution curves were fitted using double-exponential function: y = y_0_ + A_1_exp^(-DI/τ1)^ + A_2_exp^(-DI/τ2)^, where y represents ERP or EW, y_0_ is a free-fitting variable, A_1_ and A_2_ are the amplitudes, and τ_1_ and τ_2_ are the time constants of the fast (A_1_ and τ_1_) and slow (A_2_ and τ_2_) exponential components obtained by a least squares fit. The curve fitting was performed using Igor Pro 6.0 software (WaveMetrics, Inc., Portland, OR, USA). The exponential curves were then differentiated to determine the maximum slope of electrical restitution. With ventricular conduction assessments, the restitution plot fitting and differentiating were not applied owing to the flat basal relationships between the LV-to-RV conduction times and the preceding diastolic intervals (Fig 2).

### Drug infusions

In total, 32 isolated, perfused heart preparations were used in this study, in order to examine electrophysiological effects produced by flecainide, quinidine, lidocaine, and mexiletine (8 experiments in each group). For precise dosing, drug infusions were performed at a rate of 0.3 ml/min using a calibrated infusion pump, while perfusing the hearts with protein-free saline solution at a constant coronary flow rate (see above). Flecainide (1.5 μM), quinidine (5 μM), lidocaine (5 μM), and mexiletine (5 μM) (all from Sigma-Aldrich, Germany) were infused over 30 min, at concentrations close to the maximum free (i.e. protein-unbound) therapeutic plasma levels determined in cardiac patients [6, 25–30] (S1 Table).

### Data analysis

Data are expressed as mean ± standard error of the mean. One-way ANOVA was used for multiple comparisons, and paired t-tests were used to compare two data sets. *P* values less than 0.05 were considered to be significant. Original data points are given in the S1 File.

## Results

### Baseline characteristics of electrical restitution in perfused heart preparations

**Fig 3:**
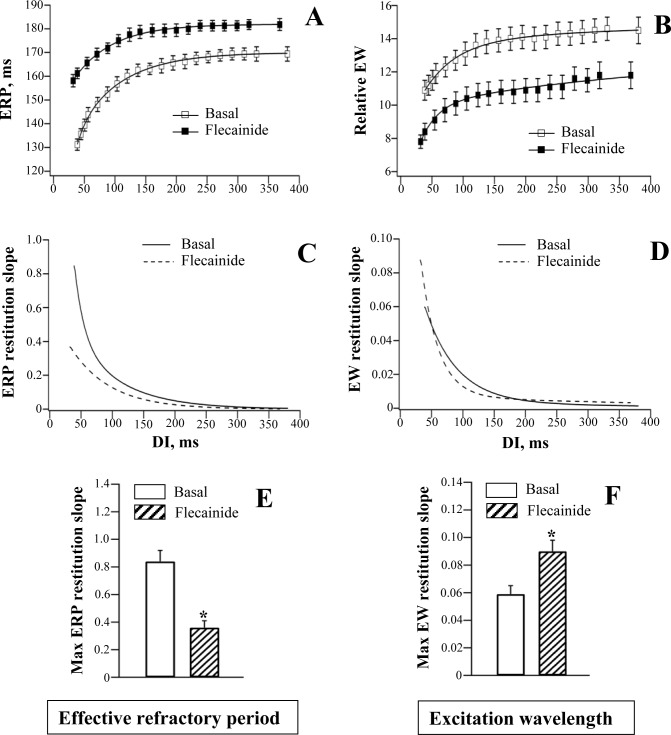
Effects of flecainide on the restitution of effective refractory period and excitation wavelength. Effective refractory periods (ERP) (panel A) and the relative excitation wavelength (EW) values (panel B) were determined at baseline and after drug infusion and plotted as a function of preceding diastolic interval (DI). The restitution curves were differentiated in order to assess changes in the restitution slope over the range of DIs used (panels C and D). Mean values of the maximum restitution slope (panels E and F) were calculated using individual measurements from each experiment. *P<0.05 vs. basal value (in panels E and F). The same figure design is used in Figs 4–6.

**Fig 4:**
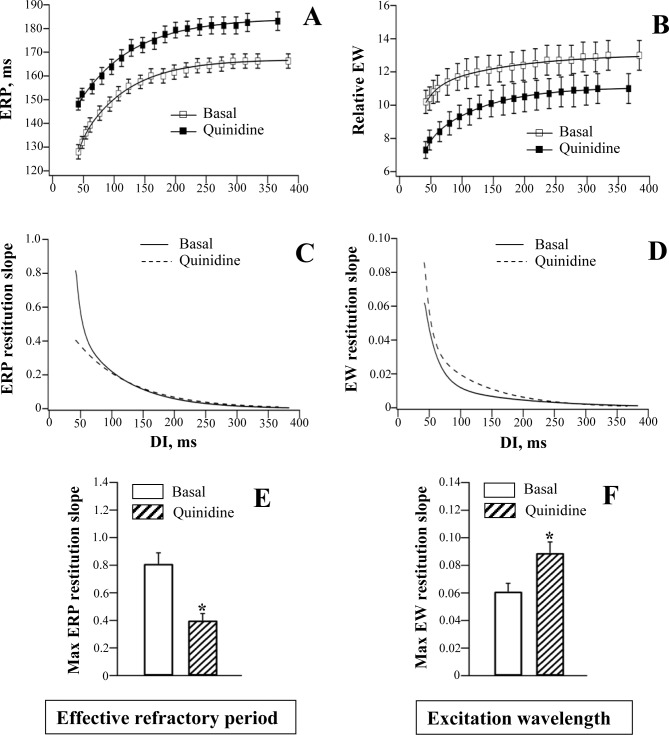
Effects of quinidine on the restitution of effective refractory period and excitation wavelength.

**Fig 5:**
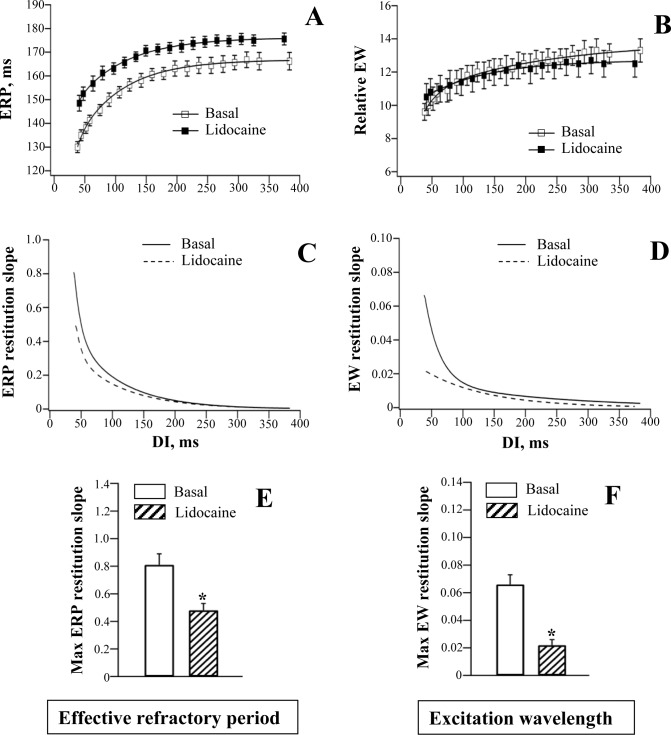
Effects of lidocaine on the restitution of effective refractory period and excitation wavelength.

**Fig 6:**
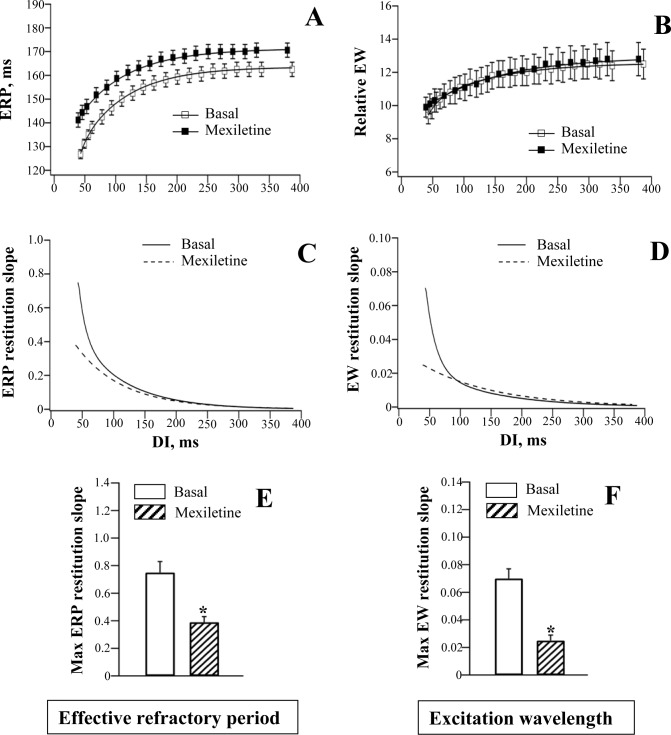
Effects of mexiletine on the restitution of effective refractory period and excitation wavelength.

Representative recordings of ventricular monophasic action potentials and assessments of ERP are shown in Fig 1, and the basal ERP restitution curves obtained in each study group are shown in Figs 3–6 (panel A, open squares). In 32 experiments, the S_1_-S_1_ reduction in a train of regular pulses from 550 ms to 169±2 ms (the minimum cycle length with 1:1 LV capture) was associated with shortening of the diastolic interval from 384±3 ms to 39±2 ms. and exponential reduction of ERP from 166±2 ms to 130±2 ms. The steepness of the ERP restitution curve was progressively increasing upon a reduction of the diastolic interval (panel C in Figs 3–6), with the averaged maximum ERP restitution slope value attained being 0.80±0.03. The LV-to-RV conduction time values (~12–13 ms) determined prior to drug infusions were relatively constant over a wide range of the diastolic intervals (Fig 2, open squares in panels A-D). Therefore, the restitution of the excitation wavelength in this setting (panels B and D in Figs 3–6) was entirely determined by the rate-dependent changes in ERP, and showed a similar time course. ANOVA revealed no between group differences in basal ERP and conduction times, as well as the maximum slopes of ERP restitution and excitation wavelength restitution.

### Flecainide and quinidine

#### ERP restitution

Flecainide and quinidine prolonged epicardial action potential duration (Table 1), and increased ERP over a wide range of stimulation intervals, thereby causing the ERP restitution curve to shift upwards (panel A in Figs 3 and 4). With both agents, the relative increase in ERP was found to be greater at the minimum (~40 ms) as compared to the maximum (~380 ms) diastolic intervals. For example, at the minimum DI, the ERP was increased upon flecainide infusion by 21% (from 131±2 ms to 158±3 ms), whereas at the maximum DI, the ERP was increased only by 8% (from 169±3 ms to 182±3 ms). With quinidine, the ERPs determined at the minimum vs. the maximum DI were increased by 16% and 10%, respectively (Minimum DI: ERP prolonged from 128±3 ms to 148±3 ms; Maximum DI: ERP prolonged from 166±3 ms to 183±4 ms).

**Table 1:** Effects of Na^+^ channel blockers on ventricular APD_90_ and QRS duration.

	Flecainide	Quinidine	Lidocaine	Mexiletine
**LV APD**_**90**_				
Basal	161±2	164±3	163±2	162±3
Drug-induced	180±3*	181±3*	162±3	167±3
**RV APD**_**90**_				
Basal	165±2	169±3	167±2	168±3
Drug-induced	177±2*	184±3*	164±4	166±4
**QRS**				
Basal	28±2	29±2	28±1	26±2
Drug-induced	35±3*	35±2*	28±2	27±2

APD_90_ and QRS values (both in ms) were determined during LV pacing at S_1_-S_1_ = 550 ms.

*P<0.05 vs. basal.

The use-dependent ERP prolongation contributed to flattening of the ERP restitution curve by flecainide (Fig 3, panels A, C and E) and quinidine (Fig 4, panels A, C and E), as evidenced by reduction in the ERP restitution slope value at short (less than 100 ms) diastolic intervals, compared to basal values. The maximum ERP restitution slope was significantly decreased by flecainide (Basal: 0.84±0.07; Flecainide: 0.36±0.05; *P* = 0.003) and quinidine (Basal: 0.81±0.08; Quinidine: 0.40±0.05; *P* = 0.01)

#### Ventricular conduction

Flecainide and quinidine increased QRS duration on ECG (Table 1), and slowed the LV-to-RV conduction (Fig 2, panels A and B). With both agents, an increase in ventricular conduction time was found to be greater at the minimum as compared to the maximum diastolic intervals. For example, at the minimum DI, the LV-to-RV conduction time was prolonged upon flecainide infusion by 75% (from 12±1 ms to 21±1 ms), whereas at the maximum DI, it was increased only by 33% (from 12±1 ms to 16±1 ms). With quinidine, the LV-to-RV conduction times determined at the minimum vs. the maximum DI were increased by 53% and 30%, respectively (Minimum DI: conduction time prolonged from 13±1 ms to 20±1 ms; Maximum DI: conduction time prolonged from 13±1 ms to 17±1 ms).

#### Excitation wavelength

In assessments of the relative excitation wavelength upon infusion of flecainide and quinidine, drug-induced conduction slowing was found to prevail over effects related to increased ERP. Indeed, despite prolonged refractoriness, the excitation wavelength values were reduced over a wide range of diastolic intervals by flecainide and quinidine (panel B in Figs 3 and 4). Importantly, owing to the use-dependent nature of conduction slowing by the Na^+^ channel blockers, the relative reduction in the excitation wavelength was larger at the minimum as compared to the maximum diastolic intervals. For example, at the minimum DI, the EW was reduced upon flecainide infusion by 28% (from 10.9±0.6 to 7.8±0.4), whereas at the maximum DI, the EW was reduced only by 18% (from 14.5±0.8 to 11.8±0.8). With quinidine, the EWs determined at the minimum vs. the maximum DI were decreased by 28% and 15%, respectively (Minimum DI: EW decreased from 10.2±0.7 ms to 7.3±0.5; Maximum DI: EW decreased from 13.0±0.9 to 11.0±0.9).

A greater reduction in EW at the minimum vs. the maximum DIs translated to the steepening of EW restitution curve by flecainide (Fig 3, panels B, D and F) and quinidine (Fig 4, panels B, D and F). The maximum EW restitution slope was significantly increased by flecainide (Basal: 0.060±0.006; Flecainide: 0.090±0.008; *P* = 0.001) and quinidine (Basal: 0.061±0.006; Quinidine: 0.089±0.008; *P* = 0.01).

### Lidocaine and mexiletine

#### ERP restitution

Lidocaine and mexiletine had no effect on epicardial action potential duration (Table 1), but moderately increased ERP (panel A in Figs 5 and 6), with effect being more pronounced at the minimum compared to the maximum diastolic intervals. With lidocaine, the ERP determined at the minimum DI was increased by 15% (from 130±2 ms to 149±3 ms; *P* = 0.0002), whereas at the maximum DI, the ERP was increased only by 6% (from 166±3 ms to 176±3 ms; *P* = 0.01). With mexiletine, the ERPs determined at the minimum vs. the maximum DI were increased by 11% and 4%, respectively (Minimum DI: ERP prolonged from 127±2 ms to 141±3 ms, *P* = 0.004; Maximum DI: ERP prolonged from 163±3 ms to 170±3 ms, *P* = 0.02).

The use-dependent ERP prolongation contributed to flattening of the ERP restitution curve by lidocaine (Fig 5, panels A, C and E) and mexiletine (Fig 6, panels A, C and E). The maximum ERP restitution slope was significantly decreased by lidocaine (Basal: 0.81±0.08; Lidocaine: 0.48±0.05; *P* = 0.004) and mexiletine (Basal: 0.75±0.08; Mexiletine: 0.39±0.04; *P* = 0.01).

#### Ventricular conduction

Lidocaine and mexiletine had no effect on either QRS duration on ECG (Table 1) or the LV-to-RV conduction delay assessed at variable stimulation intervals (panels C and D in Fig 2).

#### Excitation wavelength

In the absence of significant conduction slowing, the rate-dependent changes in the relative excitation wavelength upon infusion of lidocaine and mexiletine were largely determined by drug effects on ERP. Similar to ERP restitution, the EW restitution curve was flattened at short diastolic intervals by both agents (panels B, D and F in Figs 5 and 6). The maximum EW restitution slope was reduced by lidocaine (Basal: 0.066±0.007; Lidocaine: 0.022±0.004; *P* = 0.002) and mexiletine (Basal: 0.070±0.007; Mexiletine: 0.025±0.003; *P* = 0.01)

## Discussion

### Main findings

This study shows that the interplay between drug effects on ventricular refractoriness and conduction may result in differential changes in the rate adaptation of the excitation wavelength upon administration of class Ia (quinidine) and Ic (flecainide) compared to class Ib (lidocaine and mexiletine) Na^+^ channel blockers. Consistent with antiarrhythmic tendency, all the agents were found to use-dependently prolong ventricular refractoriness, thus flattening the ERP restitution curve. Nevertheless, when assessing the excitation wavelength dynamics, with flecainide and quinidine, the beneficial changes in ERP were reversed owing to the prominent LV-to-RV conduction slowing at short diastolic intervals, thereby leading to significantly increased steepness of the excitation wavelength restitution. In contrast, with lidocaine and mexiletine, ventricular conduction times were not changed, and therefore the maximum slope of the excitation wavelength restitution was reduced, as expected from the flattened ERP rate adaptation. Contrasting effects of the Na^+^ channel blockers on the restitution of the excitation wavelength can be considered as the contributing mechanism to the previously reported differences in clinical safety profile between class Ia and Ic vs. class Ib agents.

### Na^+^ channel blocker effects on ventricular refractoriness, conduction, and arrhythmic susceptibility

Class I antiarrhythmic agents can delay the recovery of *I*_Na_ from inactivation during ventricular repolarization, an effect which postpones the recurrence of excitability and therefore contributes to the prolonged ERP. While an increase in ERP appears to represent the “class effect” of the *I*_Na_ blockers, these agents nevertheless exhibit markedly variable efficacy in modulating ventricular conduction, which can be determined by the kinetics of drug binding to the Na^+^ channel. For example, lidocaine and mexiletine preferentially bind to the Na^+^ channels in the inactivated state (i.e., during the plateau phase), and equally reduce both the number of open channel events and the average duration of opening for each event [31–33]. These agents exhibit fast dissociation rates from their binding site in *I*_Na_ channel [8–9], meaning that drug-induced *I*_Na_ block persists during ventricular action potential, but then rapidly (within 0.09–0.18 s [34–35]) dissipates in early diastole. Consequently, class Ib agents at therapeutic concentrations produce only little or no change in ventricular conduction, at least in non-ischemic hearts [36–37]. In contrast, flecainide and quinidine preferentially bind to the open Na^+^ channels (i.e., during depolarization phase), and exhibit much slower offset kinetics. The time constant of *I*_Na_ recovery was reported to be 3–8 s for quinidine [35, 38], and 11–15 s for flecainide [35, 39]. As these values by far exceed diastolic intervals over the range of clinically relevant heart rates, the drug-induced *I*_Na_ block may persist and cumulate over successive cardiac beats, leading to the profound conduction slowing. Accordingly, in the present study, flecainide and quinidine were found to both significantly increase the QRS duration on ECG (Table 1) and use-dependently prolong the LV-to-RV conduction time (panels A and B in Fig 2), whereas lidocaine and mexiletine had no significant effect on ventricular conduction (panels C and D in Fig 2).

In studies on VT inducibility by programmed stimulation, the propensity of class I agents to suppress arrhythmia has been shown to be determined by the relations between drug effects on ERP and those on ventricular conduction [40–42]. A greater ERP prolongation at the stimulation site in the presence of minimal conduction slowing on drug administration tends to abolish re-entry and suppress the arrhythmia in patients responding to therapy, whereas more prominent conduction slowing and a lesser increase in ERP was considered to stabilize a re-entrant circuit and favor arrhythmia in patients with still inducible VT (i.e., in non-responders). These clinical findings are in line with the wavelength theory which prescribes that re-entry can be perpetuated in cardiac tissue only when the excitation wavelength, a product of ERP and conduction velocity, fits the size of the available anatomic pathway for impulse conduction [43–44]. Drug-induced reduction in the excitation wavelength, via effects on either ERP or ventricular conduction, or both, would allow the re-entry to sustain in a smaller mass of cardiac tissue, indicating a proarrhythmic effect. In contrast, an increase in the excitation wavelength can eliminate the chance to establish the re-entrant circuit in the available myocardium, indicating an antiarrhythmic action.

Although these studies can explain the mechanism for antiarrhythmic vs. proarrhythmic effects produced by class I agents at a fixed heart rate, they offer no insight into the role played by the dynamic changes in the excitation wavelength in drug-induced arrhythmogenicity. The wavelength is highly sensitive to the variations of the cardiac beating interval, and the dynamics of its rate adaptation is thought to be critical for the initiation and maintenance of reentry, and can be a factor which determines the success or failure of antiarrhythmic therapies [45–47]. With this line of reasoning, the arrhythmic safety profile of the *I*_Na_ blockers can be considered to be strongly influenced by drug effects on the kinetics of excitation wavelength restitution.

### Electrical restitution

At increased cardiac activation rates, ventricular repolarization is shortened upon a progressive reduction of the preceding diastolic interval, an effect attributed to incomplete deactivation of the outward K^+^ currents, *I*_Kr_ and *I*_Ks_ (the rapid and the slow components of the delayed rectifier, respectively). Rapid S_1_-S_1_ pacing also increases intracellular Na^+^ levels, which stimulate the reversed mode of the Na^+^-Ca^2+^ exchange and Na^+^-K^+^ pump, thus contributing to APD shortening. In addition, APD reduction in tachycardia can be partly attributed to faster inactivation of *I*_Ca_ in cardiac myocytes caused by elevated cytosolic Ca^2+^ levels [48]. With electrical restitution analysis based on plotting action potential duration as a function of the DI, the steep slope of APD rate adaptation has been shown to facilitate VF by inducing APD and Ca^2+^ transient alternans [21, 48–50], whereas flattening the electrical restitution can increase stability of the activation wavefront and prevent arrhythmia [14–15].

It should be noted, however, that the slope of the electrical restitution curve is not the only determinant of proarrhythmic tendency. The stability of the excitation wavefront can also be influenced by conduction velocity restitution, short-term cardiac memory, electrotonic effects, and Ca^2+^ cycling [15, 51–52]. These factors may dynamically interact with each other, thereby modifying arrhythmic substrate. For example, both electrotonic and memory effects can suppress arrhythmogenic APD alternans, despite a steep electrical restitution curve [52]. These complexities should be considered while interpreting Na^+^ channel blockers effects on electrical restitution. In particular, in the present study, both flecainide and quinidine were found to reduce the slope of the ERP restitution curve (panels A, C and E in Figs 3 and 4), which does not corroborate the proarrhythmic profile of these agents, as evidenced from clinical studies [3–5]. This apparent controversy raises a possibility that beneficial effects related to flattening ERP restitution can be significantly eliminated, or even reversed to proarrhythmia, owing to the drug-induced conduction slowing which translates to adverse modifications of the excitation wavelength.

### Na^+^ channel blockers and the excitation wavelength

Previous studies addressed changes in APD_90_ rate adaptation upon administration of various Na^+^ channel blockers [53–58]. However, with class I agents, the excitation wavelength restitution is likely to be more reliable than the APD_90_ restitution in assessments of the proarrhythmic tendency. First, during Na^+^ channel blocker therapy, changes in ERP may dissociate from those in APD_90_, which can be exemplified by the phenomenon of drug-induced post-repolarization refractoriness [19]. This presumes that modifications in APD_90_ rate adaptation by Na^+^ channel blocker may not necessarily be equivalent to those in ERP restitution. Second, drug-induced conduction slowing may impair the rate adaptation of the excitation wavelength and promote cardiac electrical instability, an effect which can be overlooked when assessments of arrhythmogenic tendency are solely based on measuring the APD_90_ restitution slope. Finally, the important advantage of the excitation wavelength restitution analysis is that it can give a key to discriminating Na^+^ channel blockers with antiarrhythmic and proarrhyrhmic profile of action based on the balance between the relative drug effects on ERP and ventricular conduction.

Importantly, clinical studies demonstrate that arrhythmogenic responses to Na^+^ channel blockers are often precipitated upon heart rate acceleration during exercise testing [59], thus raising a possibility that drug-induced abnormalities in the restitution of ventricular conduction and/or refractoriness, and the resulting changes in the excitation wavelength, may play a role in creating arrhythmic substrate. The present study offers a more detailed insight into the nature of this mechanism. First, because flecainide and quinidine produce a greater relative increase in the LV-to-RV conduction time compared to ERP, they reduce the absolute value of the excitation wavelength determined at a given diastolic interval (panel B in Figs 3 and 4). This tendency may potentially allow the re-entry to occur around the LV-to-RV circumference, and initiate tachyarrhythmia. Second, owing to the more pronounced conduction slowing at the short as compared to the long diastolic intervals (panels A and B in Fig 2), both flecainide and quinidine can increase the maximum slope of the excitation wavelength restitution curve (panels D and F in Figs 3 and 4). This means that for a given reduction in diastolic interval upon heart rate acceleration, the decrement in the excitation wavelength value is significantly greater in the presence of flecainide and quinidine. As a result, at the shortest DI, the relative EW was decreased by about 30% from its basal value (panel B in Figs 3 and 4) with both agents. This tendency may potentially lead to EW reduction below the minimal value necessary for stable propagation of the activation front [60]. These arguments suggest that a critical EW reduction, as facilitated by a steep EW restitution, is likely to be the most important determinant of proarrhythmia upon infusion of flecainide and quinidine.

### Limitations

Effects of Na^+^ channel blockers on ventricular refractoriness and conduction assessed in this study in normal hearts may not necessarily be identical to those induced in diseased myocardium. For example, depolarized ischemic cardiac cells may develop voltage-dependent inactivation of the Na^+^ channels, which would further accentuate depression of myocardial excitability by class I agents, and set a stage for proarrhythmia.

In the present study, the electrical restitution was analyzed by plotting ERP, ventricular conduction time, and the relative EW values vs. preceding diastolic interval. The strength of these assessments can be further increased by incorporating cardiac memory effects (e.g., via analysis of the electrical restitution portrait [51]), based on the premise that APD in a given cardiac beat is influenced by multiple preceding DIs. Whether cardiac memory effects can be modified by Na^+^ channel blockers, is uncertain, and warrants further studies.

Because ventricular conduction time is the inverse correlate of conduction velocity, the ratio between the effective refractory period and conduction time can be used as a measure of the excitation wavelength [19–22]. The direct measurements of conduction velocity rather than conduction times would likely to allow more precise evaluation of the excitation wavelength, but this would require using the multielectrode APD mapping in order to define the impulse conduction pathway, which was not performed in this study. It is noteworthy, however, that excitation wavelength assessments based on measuring ventricular conduction times in perfused hearts from different animal species were carefully validated in previous studies, and found to be reliable in detecting changes in arrhythmic tendency in the setting of hypokalemia [22], and antiarrhythmic drug infusion [19, 21]. Furthermore, in the clinical studies, the cardiac wavelength is always assessed indirectly, i.e. by correcting the ERP for QRS duration [40–41], or the local conduction delay [20], suggesting the relevance of the aforementioned approach.

In contrast to lidocaine and mexiletine, selective *I*_Na_ blockers, flecainide and quinidine can also inhibit outward K^+^ currents, such as *I*_Kr_ [61–62], which could have contributed to the prolongation of APD_90_ observed upon infusion of these agents in this study (Table 1). Excessive APD_90_ prolongation by flecainide and quinidine, in turn, can potentially promote repolarization abnormalities, including increased spatial repolarization gradients and impaired activation-to-repolarization coupling [55–56, 63]. Therefore, the mechanism by which arrhythmia can be facilitated by class Ia and Ic, as opposed to class Ib agents, is likely to be multifactorial, and not limited to their contrasting effects on the excitation wavelength dynamics.

Finally, it is worth mentioning that although class Ib Na^+^ channel blockers are regarded as safe agents, they are not widely used in contemporary cardiology practice compared to other antiarrhythmics (e.g. amiodarone). Lidocaine is not available orally, and mexiletine, its oral congener, is no longer marketed. Although lidocaine was proved to reduce the risk of VF in acute myocardial infarction [7], it does not improve mortality and may produce asystole in patients with damaged cardiac conduction system [64]. Prophylactic lidocaine is therefore no longer recommended in this setting.

## Conclusions

The steepness of the rate adaptation of the excitation wavelength in perfused guinea-pig hearts is increased by flecainide and quinidine, whereas lidocaine and mexiletine flatten the excitation wavelength restitution. These contrasting changes are likely to contribute to the differences in arrhythmic safety profile previously reported for class Ia and Ic vs. class Ib Na^+^ channel blockers in clinical studies.

## Supporting information

S1 File Original statistical data points. (XLSX)

S1 Table Therapeutic plasma levels of flecainide, quinidine, lidocaine and mexiletine and the drug concentrations used in the present study. (DOC)
