# Supplementary material for: Effects of Na+ channel blockers on the restitution of refractory period, conduction time, and excitation wavelength in perfused guinea-pig heart
Source: PLoS One. 2017 Feb 23;12(2):e0172683. doi: 10.1371/journal.pone.0172683 (PMC5322976; doi:10.1371/journal.pone.0172683)
Supplement: S1 Table — (DOC) [file pone.0172683.s002.doc]

**Supplemental Table 1.** Therapeutic plasma levels of flecainide, quinidine, lidocaine and mexiletine and the drug concentrations used in the present study.

Therapeutic plasma % protein-unbound Concentration used References

concentration fraction in this study _______________________________________________________________________________________________________________

Flecainide 0.2-1.0 µg/ml 60% 1.5 μM Conard and Ober, 1984

(or 0.5-2.5 µM)

Quinidine 2-5 µg/ml 20-30% 5 µM Kates et al., 1978;

(or 6-15 µM) Benton et al., 2000

Lidocaine 1.5-5.0 µg/ml 30% 5 µM Collinsworth et al., 1974;

(or 5-18 µM) Routledge et al., 1980

Mexiletine 0.75-2.0 µg/ml 40% 5 µM Woosley et al., 1984;

(or 4-11 µM) Pentikainen et al., 1983
